# Protective Effects of Extracts from Green Leaves and Rhizomes of *Posidonia oceanica* (L.) Delile on an In Vitro Model of the Human Blood–Brain Barrier

**DOI:** 10.3390/biology14060699

**Published:** 2025-06-14

**Authors:** Giulia Abruscato, Manuela Mauro, Marie-Christine Boucau, Vincenzo Arizza, Mirella Vazzana, Lucie Dehouck, Fabien Gosselet, Claudio Luparello, Pietra Candela

**Affiliations:** 1 Dipartimento di Scienze e Tecnologie Biologiche Chimiche e Farmaceutiche (STEBICEF), Università di Palermo, 90128 Palermo, Italy; manuela.mauro01@unipa.it (M.M.); vincenzo.arizza@unipa.it (V.A.); mirella.vazzana@unipa.it (M.V.); claudio.luparello@unipa.it (C.L.); 2 Laboratoire de la Barrière Hémato-Encéphalique (LBHE), UR 2465, Université d’Artois, F-62300 Lens, France; mchristine.boucau@univ-artois.fr (M.-C.B.); lucie.dehouck@univ-artois.fr (L.D.); fabien.gosselet@univ-artois.fr (F.G.); pietra.candela@univ-artois.fr (P.C.); 3 NBFC, National Biodiversity Future Center, 90133 Palermo, Italy

**Keywords:** seagrass, blood–brain barrier, VCAM-1, ICAM-1, NLRP3, tight junctions

## Abstract

*Posidonia oceanica* (L.) Delile, a Mediterranean seagrass, is rich in bioactive compounds with anti-inflammatory potential. While marine-derived molecules are increasingly studied, their direct effects on blood–brain barrier (BBB) integrity under inflammatory conditions remain largely unexplored. This study evaluated the ability of aqueous extracts from its green leaves (GLEs) and rhizomes (REs) to protect the BBB using a human in vitro model consisting of brain-like endothelial cells co-cultured with brain pericytes. The model was exposed to TNFα, with or without GLEs or REs. We assessed NO production, endothelial permeability, expression of IL-6, NLRP3, ICAM-1, VCAM-1, CLAUDIN-5, and VE-CADHERIN, and the localization of junctional proteins. TNFα increased NO and IL-6 release, upregulated ICAM-1, VCAM-1, and NLRP3, and impaired BBB integrity by altering junctional protein levels and distribution. Co-treatment with GLEs or REs reduced the production of NO, the expression of NLRP3 and adhesion molecules and restored tight and adherens junction integrity. IL-6 levels remained unaffected. These findings suggest that *P. oceanica*’s extracts may help preserve BBB function and mitigate inflammation-induced damage. While further studies are needed to assess their bioavailability and in vivo efficacy, these natural compounds represent promising candidates for developing preventive strategies against neuroinflammatory disorders.

## Simple Summary

As an endemic species, *Posidonia oceanica* is extensively distributed and commonly found throughout the Mediterranean region. This study aimed to explore new natural agents capable of preserving the integrity of the human blood–brain barrier (BBB) under inflammatory stress. Using an in vitro BBB model, we evaluated the anti-inflammatory and protective effects of aqueous extracts from green leaves (GLEs) and rhizomes (REs) of *P. oceanica.* Our results show that GLEs and REs reduce nitric oxide production and inflammatory markers while preserving the structural cohesion of the endothelial barrier. Since BBB integrity is essential for brain homeostasis and its disruption contributes to various neurological diseases, these findings suggest that *P. oceanica*’s extracts may represent promising candidates for further research on strategies to prevent or mitigate neuroinflammatory disorders.

## 1. Introduction

The marine habitat, renowned for its extraordinary biodiversity, remains a largely untapped reservoir of primary and secondary metabolites [1]. These bioactive compounds, synthesized by marine animals, plants, and micro-organisms, exhibit remarkable biological properties and play key roles in chemical communication networks that support adaptation and survival in this challenging environment.

*Posidonia oceanica* (L.) Delile (1813; Liliopsida, Najadales: Posidoniaceae) is an endemic angiosperm of the Mediterranean Sea. Its anatomical features include long ribbon-shaped leaves, rhizomes (modified stem structures), and short roots, each exhibiting distinct phytochemical compositions [2,3]. These are extensive meadows, which support the reproductive and juvenile stages of a variety of marine species and serve as significant biological indicators for monitoring marine ecosystem health [4]. The consistent transport of floating detached leaves, rhizomes, and roots to coastal areas leads to the formation of solid “banquettes”, that significantly contribute to the accumulation of organic waste that dehydrates and decomposes over time. Therefore, the valorization of fresh and dead *P. oceanica*’s residues as a sustainable biomass source has been the focus of numerous studies, leading to their application in areas such as biocomposites, livestock nutrition, composting, biofuel production, and the extraction of bioactive molecules for biomedical use. Historically, *P. oceanica* has a long-standing role in folk medicine, having been traditionally used to treat a variety of ailments including diabetes, throat infections, hypertension, acne, and colitis [5,6,7,8]. Recent in vitro studies have shown that extracts from green leaves (GLEs) and rhizomes (REs) exhibit strong anti-cancer and anti-metastatic effects against hepatocarcinoma cells, mainly by promoting apoptosis and impairing autophagic flux. Moreover, *P. oceanica*’s extracts have been found to reduce gelatinase levels in fibroblastoma and neuroblastoma cell cultures [2,9,10,11]. Both in vitro and in vivo studies have also demonstrated their anti-glycation activity and ability to lower glucose levels [12,13,14].

Extracts from *P. oceanica* provide anti-inflammatory and protective benefits by inhibiting NF-κB activation through the modulation of ERK1/2 and AKT signaling pathways, resulting in lowered levels of iNOS and COX-2 expression, along with diminished production of reactive oxygen species and nitric oxide (NO) [15]. Moreover, these extracts have proven to be effective in mitigating UV-induced photodamage and reducing free radical generation in fibroblasts [16].

Notably, *P. oceanica*’s extracts show promising potential for neurological applications, especially given their documented ability to cross the blood–brain barrier (BBB) [17,18,19]. The BBB, composed mainly of endothelial cells (ECs) connected by tight junctions (TJs), regulates molecular traffic and preserves brain homeostasis [20]. Under inflammatory conditions, upregulation of adhesion molecules such as ICAM-1 and VCAM-1 promotes immune cell infiltration and disrupts junctional integrity, leading to increased BBB permeability [21]. Although marine-derived compounds, including flavonoid-rich extracts from *P. oceanica*, are known for their anti-inflammatory, antioxidant, and neuroprotective properties [22,23], their direct effects on BBB integrity, particularly under inflammatory stress, remain insufficiently clarified. This represents a critical gap, especially in the context of neuroinflammatory diseases such as Alzheimer’s, Parkinson’s, and multiple sclerosis, where BBB dysfunction is a significant hallmark [21,24].

Based on our previous findings showing the anti-inflammatory activity of *P. oceanica*’s GLEs and REs [25], we intended to examine their ability to preserve BBB integrity in an inflammatory context. We used a human in vitro BBB model composed of ECs derived from hematopoietic stem cells co-cultured with human brain pericytes [26]. These ECs acquire key BBB features and are designated as brain-like endothelial cells (BLECs) [27,28]. Inflammation was induced by TNFα, a cytokine commonly associated with BBB impairment in vivo [21]. Although further research is needed to evaluate the oral bioavailability and brain penetration of these marine compounds, our model enables the assessment of their direct protective effects on the BBB. This study lays the groundwork for investigating the efficacy of *P. oceanica*’s extracts as potential therapeutic agents for neuroinflammatory disorders.

## 2. Materials and Methods

### 2.1. Preparation of GLEs and REs

GLEs and REs from *P. oceanica* were prepared following the established protocols described in [2]. Briefly, the green leaves and rhizomes were collected by scuba diving off the coast of the Gulf of Palermo (Sicily, Italy). After being thoroughly washed, each sample was ground individually in a mortar with liquid nitrogen. The powders produced were resuspended in 2M acetic acid supplemented with a 1:200 dilution of antiprotease cocktail (P8340, Sigma, St. Louis, MO, USA), and then homogenized, sonicated, and centrifuged at 15,500 rpm for 20 min at 4 °C. The supernatants were subjected to filter-sterilization, lyophilized, and then reconstituted in the minimum necessary volume of sterile distilled water and stored at −20 °C for later use.

Both preparations underwent analysis of their polyphenol content and proteomic profile, with the polyphenol composition being characterized through a qualitative–quantitative HPLC approach, as outlined in Table 1 [2].

### 2.2. Cell Cultures

The mouse macrophage cell line RAW 264.7 (RRID:CVCL_0493), taken from laboratory stocks, was grown in glutamine-containing DMEM (Gibco^TM^, Fisher Scientific, Segrate, Italy) supplemented with 10% heat-inactivated fetal bovine serum (Sigma) and antibiotics (100 U/mL penicillin and 100 µg/mL streptomycin; Capricorn Scientific GmbH, Ebsdorfergrund, Germany) at 37 °C under 5% CO_2_ in a humidified atmosphere.

### 2.3. Human BBB Model

The in vitro model of the human BBB was established using BLECs according to the protocol developed by Cecchelli et al. [28]. Human hematopoietic stem cells were isolated from umbilical cord blood, as described by Pedroso et al. [29], and subsequently differentiated into ECs. The human brain pericytes (hBPs), obtained from an external collaborator (Department of Neurology and Clinical Neuroscience, Yamaguchi University, Japan) [30], were characterized for pericyte markers as described by Menaceur et al. [26] and were cultured in an appropriate medium for pericytes under standard conditions. The ECs were co-cultured with human brain pericytes (hBPs) at 37 °C with 5% CO_2_ in the appropriate medium to create a functional BBB model, as previously described [30]. The medium was renewed every two days, and after a total of six days of incubation, the model was set for treatment procedures. At this stage, the BLECs had formed a functional barrier, dividing into apical (blood side) and basolateral (brain side) compartments.

HBPs and CD34^+^-hematopoietic stem cells were regularly checked for mycoplasma contamination, and short tandem repeat (STR) analysis confirmed the absence of cross-contamination.

### 2.4. Treatments

GLEs and REs were prepared in either complete DMEM (for RAW 264.7 cells) or a basal EC growth medium supplemented with 0.1% BSA (for the BBB). Macrophages were exposed to 0.1 µg/mL LPS (*Escherichia coli*, O55:B5 Sigma), either with or without GLEs or REs at different concentrations (0.25, 0.5, 1, 5, and 10 µg/mL for GLEs and 0.025, 0.05, and 0.1 µg/mL for REs), for 24 h. For the BBB, the extracts were applied to the luminal compartment (blood side), and after 30 min of exposure, TNF-α (SRP3177; Sigma) was applied at a concentration of 5 ng/mL to the same compartment for an additional 24 h, either with or without GLEs and REs at different concentrations (10 µg/mL for GLEs and 0.1 µg/mL for REs) to induce an inflammatory state.

### 2.5. Evaluation of NO Release in Culture Media

To assess the nitric oxide (NO) scavenging ability of GLEs and REs, we measured nitrite levels, a stable NO metabolite in two experimental models: LPS-stimulated RAW 264.7 macrophages (150,000 cells/ well in 12-well plates) and a human in vitro BBB model exposed to TNFα. Cell culture supernatants (for macrophages) and samples from the luminal compartment (for the BBB) were collected at the end of the treatments.

The quantification of nitrite was performed using the Griess reagent (Biotium, Fremont, CA, USA) following the manufacturer’s instructions. Briefly, samples were transferred to 96-well plates and incubated with the reagent for 30 min at room temperature, protected from light. Absorbance was measured at 548 nm using a microplate reader. The amount of NO released under each experimental condition was expressed as the ratio of the optical density (O.D.) of treated samples to that of untreated controls [31].

### 2.6. Evaluation of BBB Permeability

The effects of GLEs and REs on BBB monolayer integrity following TNFα exposure were evaluated by measuring the endothelial permeability coefficient (Pe) of sodium fluorescein (NaFlu), a small hydrophobic molecule that exhibits a low penetration rate across the BBB under physiological conditions, as previously described by Dehouck et al. [27]. Essentially, after 24 h of treatment, the BLECs-containing inserts were transferred to wells containing HEPES-buffered Ringer’s solution (RH), and NaFlu was added to the luminal compartment, where its Pe serves as an indicator of the monolayer’s integrity. Every 20 min the inserts were transferred to a new well that contained 500 µL of warmed RH, and this transfer process was carried out for a total of 1 h. At the end of the incubation, the inserts were removed, and aliquots of the donor solution were collected both at the start and conclusion of the experiment. Pe was assessed by measuring fluorescence at 490/525 nm using a microplate reader (Synergy H1^®^, BioTek). To determine the cleared volume, the final concentration of NaFlu in the receiving compartment was divided by the initial concentration in the donor compartment and then divided by the total duration of the experiment (60 min) to calculate the permeability surface area product (PS, µL/min). The analysis included permeability assessments for both cell-free (PSf) filters and filters that contained cells (PSt).

1/PSe = 1/Pst − 1/Psf

The permeability surface area product of the EC monolayer (PSe, µL/min) was calculated, and subsequently the PeNaFlu values related to BLECs (Pe, cm/min) were obtained by dividing the PSe value by the filter’s surface area (1.12 cm^2^) and using the formula “PeNaF = PSe/S”.

### 2.7. Fluorescence Microscopy

After 24 h of treatment with the sole TNFα or co-treatment with TNFα and either GLEs or REs, BLECs were rinsed with PBS for 5 min, then fixed and permeabilized as described by Versele et al. [21]. After blocking with Sea Block Blocking Buffer (37527; Thermo Fisher, Rockford, IL, USA) for 30 min, BLECs were incubated for 1 h with one of the following rabbit primary antibodies in a 5% Sea Blocking Buffer in PBS-CMF at room temperature: anti-CLAUDIN-5 (34-1600, Thermo Fisher; working dilution 1:200), anti VE-CADHERIN (ab33168, Abcam, Cambridge, UK; working dilution: 1:400). After additional washes, the cells were incubated with the fluorochrome-conjugated secondary polyclonal antibodies (Goat anti-Rabbit Alexa Fluor 488, A11034, Molecular Probes, Eugene, OR, USA) diluted 1:500 in 5% sea blocking buffer in PBS-CMF for 30 min in the dark and at room temperature. After completing the final washing procedures, the filters were placed on glass slides and mounted under coverslips with ProLong Gold Antifade Mountant^®^ containing DAPI (Thermo Fisher). Images were acquired and analyzed in a Axio Imager A2^®^ fluorescence microscope (Leica Microsystems, Wetzlar, Germany) and processed with ImageJ 1.54g software.

### 2.8. mRNA Expression Analysis

Analysis of mRNA expression was performed by RT-qPCR, as reported by Abruscato et al. [2] and Versele et al. [21]. Essentially, total RNA from control and treated BLECs of the BBB were extracted using the NucleoSpin^®^ RNA/Protein mini kit (Macherey-Nagel, Dueren, Germany) according to the manufacturer’s instructions. The amount and purity of extracted mRNA was assessed using Synergy H1 spectrophotometer (Biotek, Colmar, France) measuring the absorbance at 260, 280, and 320 nm. Reverse transcription was performed using iScript^TM^ reverse transcription supermix (Bio-Rad, Hercules, CA, USA). SYBR Green-based qPCR was performed in 96-well plates on triplicate samples. Differential gene expressions in BLECs were analyzed using the SsoFast^TM^ Evagreen SuperMix (Bio-Rad) and the primer pairs reported in [32,33,34] in a CFX96 Real-Time System thermal cycler (Bio-Rad). Gene expression levels were estimated using the 2^−ΔΔCt^ method and normalized to *GAPDH* as a reference gene.

### 2.9. Enzyme Linked Immunosorbent Assay (ELISA)

The amount of IL-6 released in the cell-free supernatants of control and treated BLECs was estimated using the Human IL-6 ELISA kit (Ab178013, Abcam), according to the manufacturer’s instructions.

### 2.10. Western Blot

After treatments, the cells were collected in a RIPA buffer (Millipore, Burlington, MA, USA) supplemented with protease and phosphatase inhibitor cocktails (Sigma Aldrich), and the cell lysates were then centrifuged at 10,000 rpm for 10 min at 4 °C. Protein concentration was measured using a Bradford assay (Bio-Rad) according to the manufacturer’s instructions. Equal amounts of protein lysates (20 µg) were combined with a Laemmli reagent (Bio-Rad), heated at 95 °C for 5 min, and subjected to SDS-PAGE on 4–15% acrylamide gels (Bio-Rad) at 200 V for 45 min. The separated proteins were transferred to nitrocellulose membranes (GE Healthcare, Amersham, UK) at 100 V for 1 h. The membranes were then blocked with TBS-T containing 1% Tween 20 and 5% skim milk for 1 h, followed by incubation with primary antibodies at 4 °C overnight, except for β-actin. The rabbit and mouse primary antibodies used to probe the blots were anti-CLAUDIN-5 (GTX49370, Genetex, Irvine, CA, USA; working dilution 1:1000), anti-VE-CADHERIN (ab33168; working dilution 1:1000), anti-ICAM-1 (Ab53013; working dilution 1:2000), anti-VCAM-1 (PA5-80213, Invitrogen; working dilution 1:1000), anti-NLRP3 (Ab263899; working dilution 1:1000), and as an internal control, either anti-GAPDH (GTX627408, Genetex; working dilution 1:20,000) or anti-actin (A5441, Sigma; working dilution 1:20,000). After reaction with the peroxidase-conjugated secondary antibodies (P0447 and P0448, Dako/Agilent Technologies, Inc., Santa Clara, CA, USA; working dilution 1:5000 for P0447 and 1:8000 for P0448) at room temperature for 1 h and incubation with an enhanced chemiluminescence reagent (ECL, Cytiva^TM^, Amersham, UK), the bands were detected using the Azure c600 Western immunoblotting imaging system (Azure Biosystems, Dublin, Ireland). Protein band intensities were quantified using ImageJ software, and the data were normalized to the intensity of the internal control band.

### 2.11. Statistics

All the results are representative of at least three independent experiments performed in triplicate. Data are presented as mean ± SEM. One-way variance analysis (ANOVA) with post hoc comparison tests were performed. For statistical analysis, the GraphPad 9 Prism software (GraphPad Software, San Diego, CA, USA) was used.

## 3. Results

### 3.1. Effect of GLEs and REs on NO Production by Inflamed RAW 264.7 Cells and BLECs

Nitric oxide (NO), when produced in excess, plays a key role in the inflammatory response. Therefore, compounds that can attenuate its production are considered promising candidates for managing inflammatory conditions [34].

**Figure 1:**
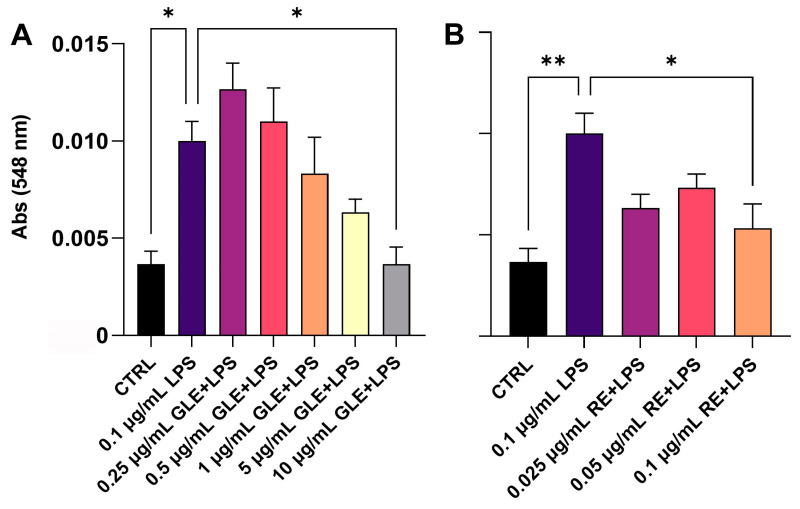
Effect of 0.1 µg/mL LPS, alone and in co-treatment with different concentrations of GLEs (**A**) and REs (**B**), on NO release in the culture media of inflamed RAW 264.7 cells, quantified with the Griess reaction. Each bar is representative of at least three independent experiments performed in triplicate. Values are expressed as the mean ± SEM. One-way ANOVA followed by Tukey’s multiple comparison post test was used. * *p* < 0.05; ** *p* < 0.01.

As a first step, we assessed whether *P. oceanica*’s GLEs and REs could reduce NO release by LPS-stimulated RAW 264.7 macrophages (0.1 µg/mL LPS). Concentrations previously identified as non-cytotoxic [25] were selected for the assay. As shown in Figure 1A,B, both GLEs and REs significantly reduced NO production in a dose-dependent manner after 24 h of treatment.

To extend these findings to the BBB context, we evaluated the extracts in the human in vitro BBB model. Inflammation was induced by applying 5 ng/mL TNFα to the luminal compartment for 24 h. Based on the data from macrophage studies, we chose concentrations of 10 µg/mL GLEs and 0.1 µg/mL REs to address the increased complexity and lower sensitivity of endothelial cells compared to macrophages.

**Figure 2:**
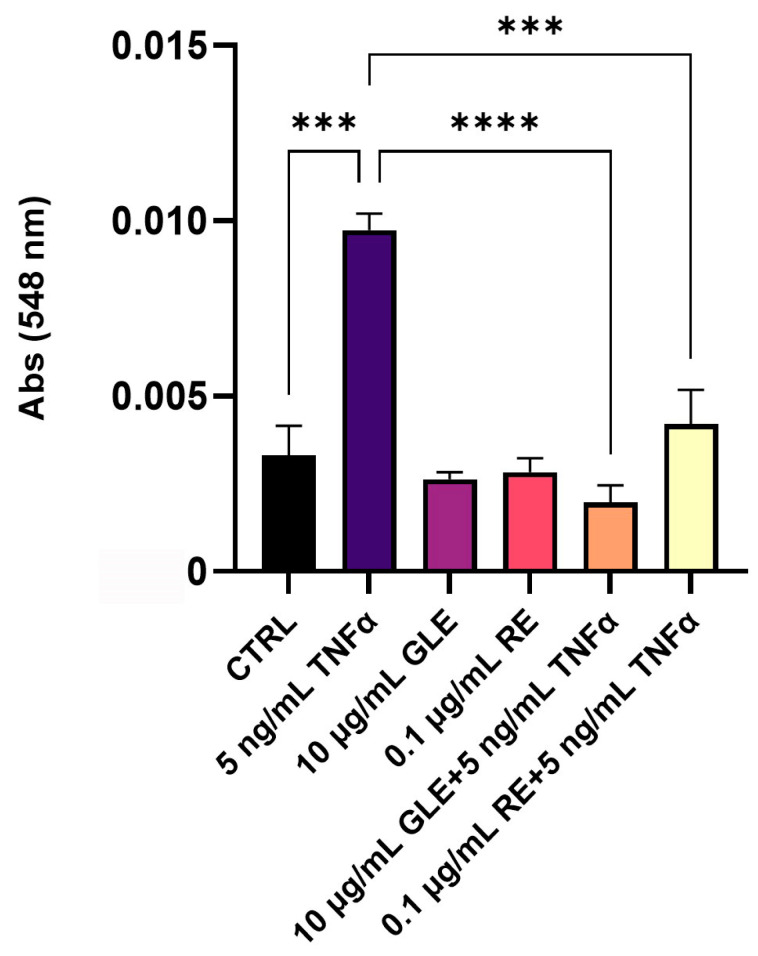
Effect of 10 µg/mL GLEs and 0.1 µg/mL REs, alone and in co-treatment with 5 ng/mL TNFα, on NO release in the culture media of BLECs, quantified with the Griess reaction. Each bar is representative of at least three independent experiments performed in triplicate. Values are expressed as the mean ± SEM. One-way ANOVA followed by Tukey’s multiple comparison post test was used. *** *p* < 0.001; **** *p* < 0.0001.

As shown in Figure 2, TNFα stimulation led to a ~1.9-fold increase in NO levels in the BLEC culture medium. Co-treatment with GLEs or REs significantly attenuated this effect, reducing NO production to near-baseline levels (−5.1-fold and −2.3-fold vs. TNFα, respectively). Importantly, treatment with GLEs or REs alone did not significantly alter NO levels compared to the untreated control.

### 3.2. Impact of GLEs and REs on Inflammatory Markers IL-6 and NLRP3

**Figure 3:**
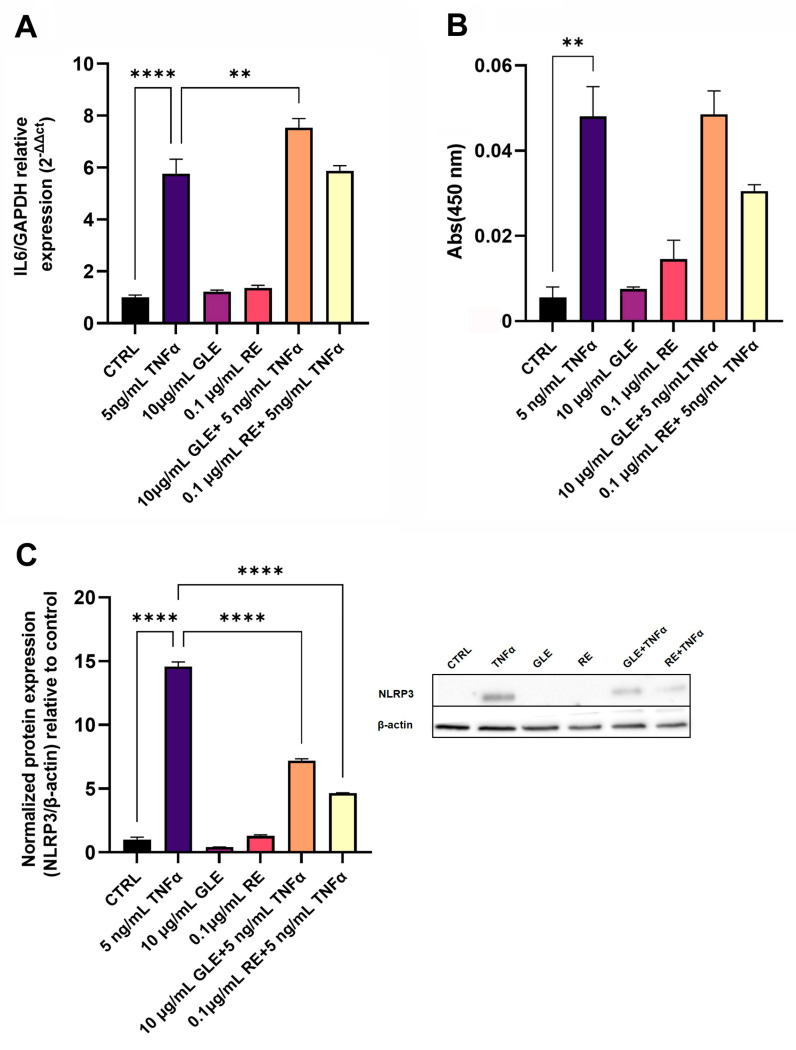
Effect of 10 µg/mL GLEs and 0.1 µg/mL REs, alone and in co-treatment with 5 ng/mL TNFα, on the IL-6 mRNA expression level and protein amount secreted in the culture medium of BLECs, determined by qRT-PCR (**A**) and ELISA (**B**) respectively, and on the NLRP3 protein expression level (**C**). The original Western Blot is included in the Supplementary Materials. Each bar is representative of at least three independent experiments performed in triplicate. Values are expressed as mean fold change ± SEM compared to the control. One-way ANOVA followed by Tukey’s multiple comparison post test was used. ** *p* < 0.01; **** *p* < 0.0001.

We evaluated the expression and release of the pro-inflammatory cytokine IL-6 under all experimental conditions. As shown in Figure 3, TNFα treatment upregulated the IL-6 mRNA level by about 5.76-fold and IL-6 protein release by about 8.7-fold compared to the controls. GLE and RE treatments maintained the transcription level and cytokine release rate in the extracellular medium similar to the control. On the other hand, co-exposure to TNFα and GLEs induced an increase in the IL-6 mRNA level by about 1.3-fold compared to TNFα, while co-exposure to TNFα and REs did not alter IL-6 mRNA and protein levels compared to TNFα alone. Regarding the inflammasome component NLRP3, TNFα markedly upregulated its protein expression by 14.56-fold. Co-treatment with GLEs or REs significantly reduced NLRP3 expression by 2.02-fold and 3.14-fold, respectively, compared to TNFα treatment.

### 3.3. Effect of GLEs and REs on the Expression of ICAM-1 and VCAM-1 in Inflamed BLECs

ICAM-1 and VCAM-1 are key adhesion molecules expressed by ECs, typically suppressed under physiological conditions and markedly upregulated during inflammation. Their expression facilitates leukocyte adhesion and transmigration [32] and contributes to calcium-mediated cytoskeletal remodeling [33], making them well-established molecular markers of BBB inflammation. To further assess the anti-inflammatory potential of GLEs and REs, we evaluated ICAM-1 and VCAM-1 expression at both mRNA and protein levels in BLECs exposed to TNFα, with or without co-treatment.

**Figure 4:**
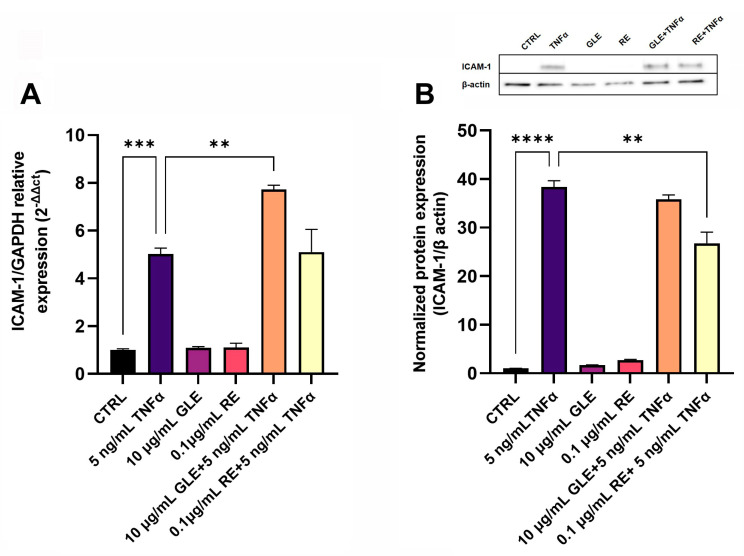
Effect of 10 µg/mL GLEs and 0.1 µg/mL REs, alone and in co-treatment with 5 ng/mL TNFα, on mRNA and protein expression levels of the cell adhesion molecule ICAM-1 in BLECs, determined by qRT-PCR (**A**) and Western blot (**B**), respectively. The original Western Blot is included in the Supplementary Materials. Each bar is representative of at least three independent experiments performed in triplicate. Values are expressed as mean fold change ± SEM compared to the control. One-way ANOVA followed by Tukey’s multiple comparison post test was used. ** *p* < 0.01; *** *p* < 0.001; **** *p* < 0.0001.

As shown in Figure 4A, after 24 h of exposure to TNFα, the level of ICAM-1 mRNA was increased by about 5-fold. A condition comparable to the control was found after treatment with the sole GLEs or REs. The co-exposure to GLEs and TNFα increased ICAM1 expression by about 1.54-fold, while no statistically significant modification in transcript levels was observed with REs/TNFα co-treatment, compared to the sole TNFα. On the other hand, as shown in Figure 4B, after 24 h of exposure to TNFα, the amount of the ICAM-1 protein was upregulated by about 38-fold. This increased level was downregulated by about 1.4-fold following co-treatment with REs, whereas no change was observed when cells were co-exposed to GLEs. The results obtained after treatments with the sole GLEs or REs were comparable to those of the control.

**Figure 5:**
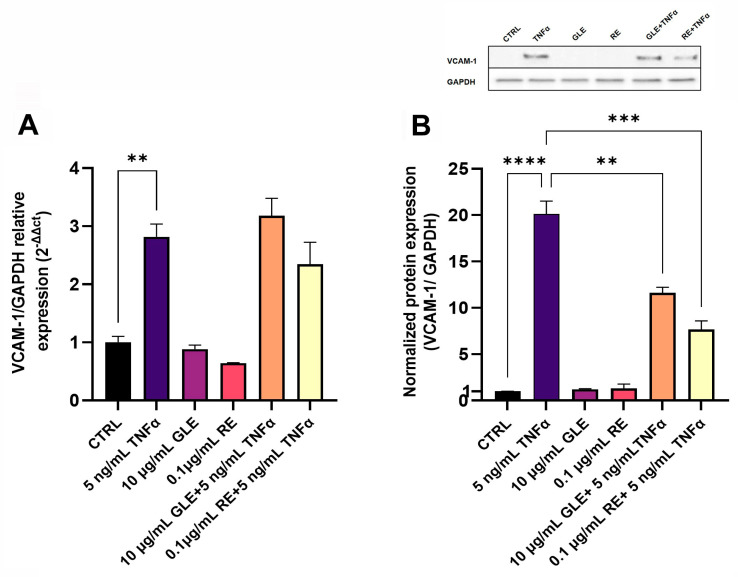
Effect of 10 µg/mL GLEs and 0.1 µg/mL REs, alone and in co-treatment with 5 ng/mL TNFα, on mRNA and protein expression levels of the cell adhesion molecule VCAM-1 in BLECs, determined by qRT-PCR (**A**) and Western blot (**B**), respectively. The original Western Blot is included in the Supplementary Materials. Each bar is representative of at least three independent experiments performed in triplicate. Values are expressed as mean fold change ± SEM compared to the control. One-way ANOVA followed by Tukey’s multiple comparison post test was used. ** *p* < 0.01; *** *p* < 0.001; **** *p* < 0.0001.

Concerning VCAM-1, as shown in Figure 5A after 24 h of exposure to TNFα, its mRNA level was increased by about 2.8-fold compared to the control. Co-treatments with GLEs or REs and TNFα did not alter the mRNA levels in a statistically significant manner. Furthermore, conditions comparable to the control were found after treatment with the sole GLEs or REs. On the other hand, after 24 h of exposure to TNFα, the VCAM-1 protein level was increased by about 20.1-fold, and a significant reduction by about 1.7 and 2.6-fold was observed after co-treatment with GLEs or REs, respectively (Figure 5B). The results obtained after treatments with the sole GLEs or REs were comparable to those of the control.

### 3.4. Effect of GLEs and REs on the Permeability of Inflamed BLEC Monolayer

To investigate the potential protective effects of GLEs and REs on BBB permeability following TNFα-induced inflammation, we monitored the physical integrity of the BLEC monolayer by measuring the NaFlu passage rate from the luminal to the abluminal compartment and calculating the corresponding Pe.

**Figure 6:**
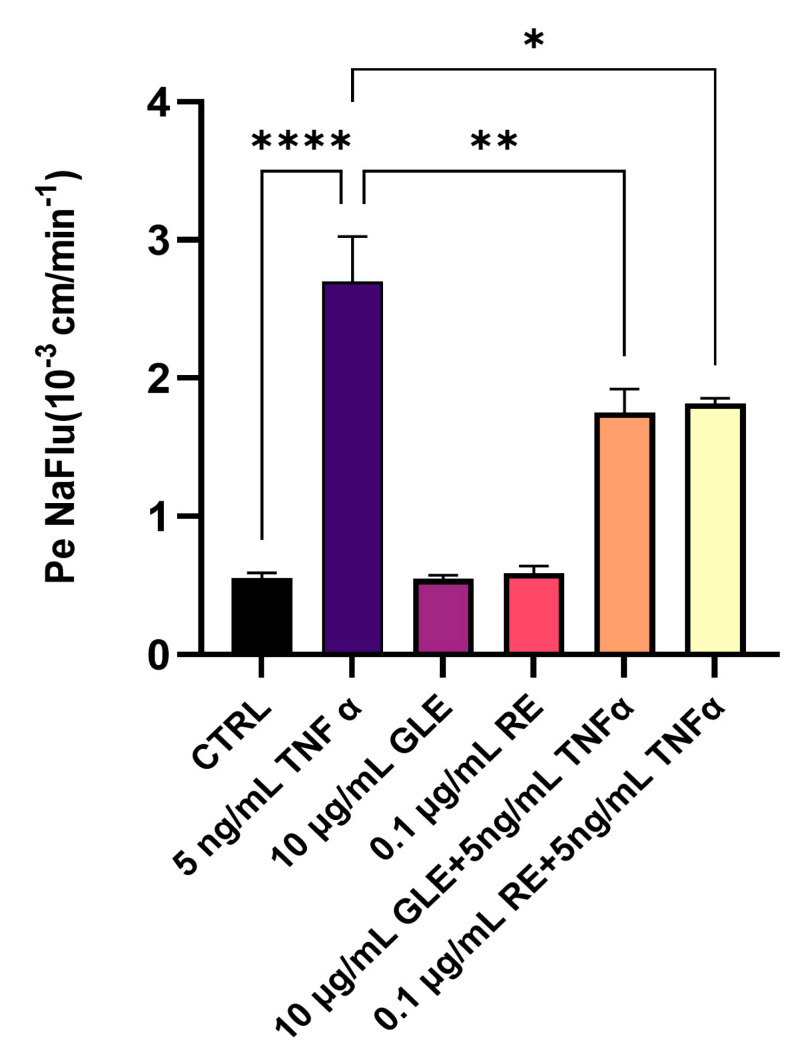
Effect of 10 µg/mL GLEs and 0.1 µg/mL REs, alone and in co-treatment with 5 ng/mL TNFα, on the permeability of the BLEC monolayer, determined by measuring the Pe of NaFlu. Each bar is representative of at least three independent experiments performed in triplicate. Values are expressed as the mean ± SEM. One-way ANOVA followed by Tukey’s multiple comparison post test was used. * *p* < 0.05; ** *p* < 0.01; **** *p* < 0.0001.

As shown in Figure 6, treatments with GLEs or REs alone did not significantly alter BBB permeability, with Pe values similar to the untreated control (control: 0.55 × 10^−3^ cm·min^−1^; GLEs: 0.54 × 10^−3^ cm·min^−1^; REs: 0.59 × 10^−3^ cm·min^−1^), indicating that the extracts do not compromise barrier integrity in the absence of inflammation. Exposure to TNFα (5 ng/mL for 24 h) significantly increased monolayer permeability, with Pe reaching approximately 2.7 × 10^−3^ cm·min^−1^, confirming BBB disruption under inflammatory conditions. The protective effects of 25-hydroxycholesterol and pioglitazone against TNFα-induced BBB disruption have been previously demonstrated [34,35]; therefore, these compounds were not included as positive controls in the current experimental design. Co-treatment with GLEs or REs significantly reduced TNFα-induced permeability, with Pe values decreasing to 1.75 × 10^−3^ cm·min^−1^ (TNFα + GLEs) and 1.81 × 10^−3^ cm·min^−1^ (TNFα + REs), indicating that both extracts partially preserve endothelial barrier integrity and mitigate inflammatory insults.

### 3.5. Effect of GLEs and REs on the Expression and Localization of CLAUDIN-5 and VE-CADHERIN

The integrity and functionality of the BBB are regulated by various junctional complexes that control its permeability. TNFα is known to induce alterations in BBB permeability associated with changes in the architecture of TJ and AJ proteins, as widely reported in the literature [21]. We therefore investigated whether GLEs and REs could counteract TNFα-induced changes in the expression levels and cellular localization of CLAUDIN-5 and VE-CADHERIN, molecular constituents of these intercellular junctions, using qRT-PCR, Western blot, and immunostaining assays.

**Figure 7:**
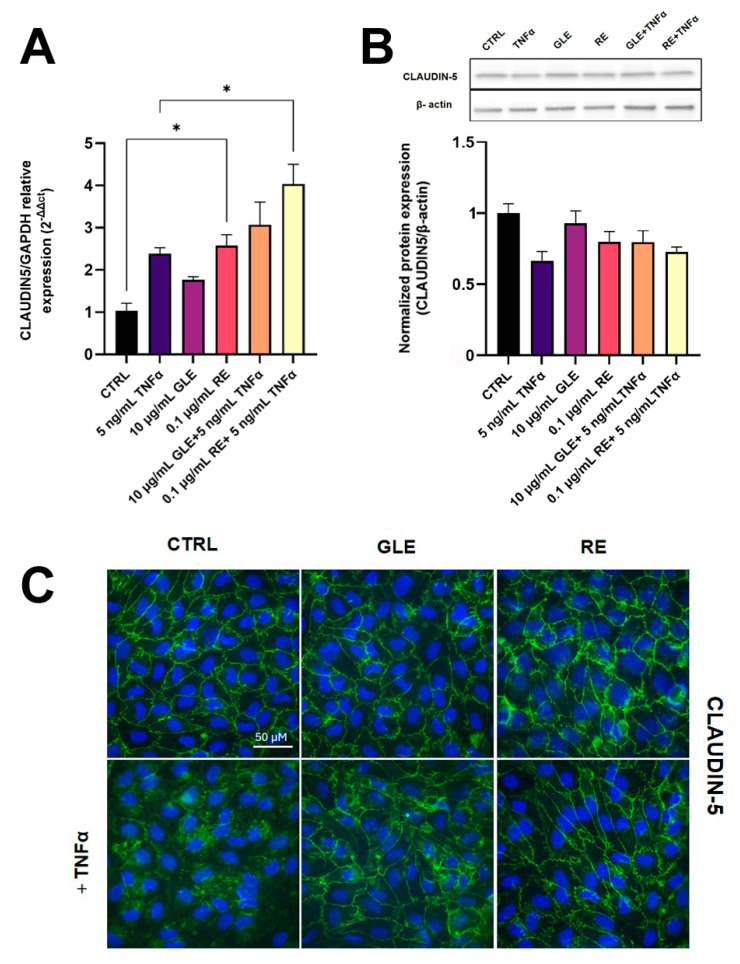
Effect of 10 µg/mL GLEs and 0.1 µg/mL REs, alone and in co-treatment with 5 ng/mL TNFα, on the expression and localization of the TJ protein CLAUDIN-5 in BLECs, determined by qRT-PCR (**A**), Western blot (**B**) and immunofluorescence microscopy with nuclear counterstaining using DAPI (**C**). The original Western Blot is included in the Supplementary Materials. Each bar is representative of at least three independent experiments performed in triplicate. Values are expressed as mean fold change ± SEM compared to the control. One-way ANOVA followed by Tukey’s multiple comparison post test was used. * *p* < 0.05.

As shown in Figure 7A, the transcriptional level of CLAUDIN-5 significantly increased after exposure to REs alone by about 2.57-fold compared to control. The sole co-treatment with REs and TNFα resulted in an increment of CLAUDIN-5 mRNA expression by about 1.69-fold compared to TNFα alone. Interestingly, treatment with REs alone also induced an about 2.57-fold upregulation of the CLAUDIN-5 transcript level compared to the control. Western blot analysis indicated that following TNFα treatment, CLAUDIN-5 protein expression levels were decreased by about 0.4-fold compared to the control. In contrast, the co-administration of GLEs and REs with TNFα exhibited a non-significant tendency for the upregulation of CLAUDIN-5 protein compared to the treatment with TNFα alone (Figure 7B). Changes in CLAUDIN-5 localization were studied by immunofluorescence analysis. As shown in Figure 7C, treatment with TNFα alone led to the expected delocalization of the TJ protein to cytosolic compartments. On the other hand, co-treatment with TNFα and GLEs or REs restored the correct localization of the protein to cell boundaries. Treatments with GLEs or REs alone maintained the localization of the protein comparable to control conditions. These findings indicate that GLEs and REs can counteract TNFα-induced derangement in protein localization, thereby potentially preserving BBB integrity.

**Figure 8:**
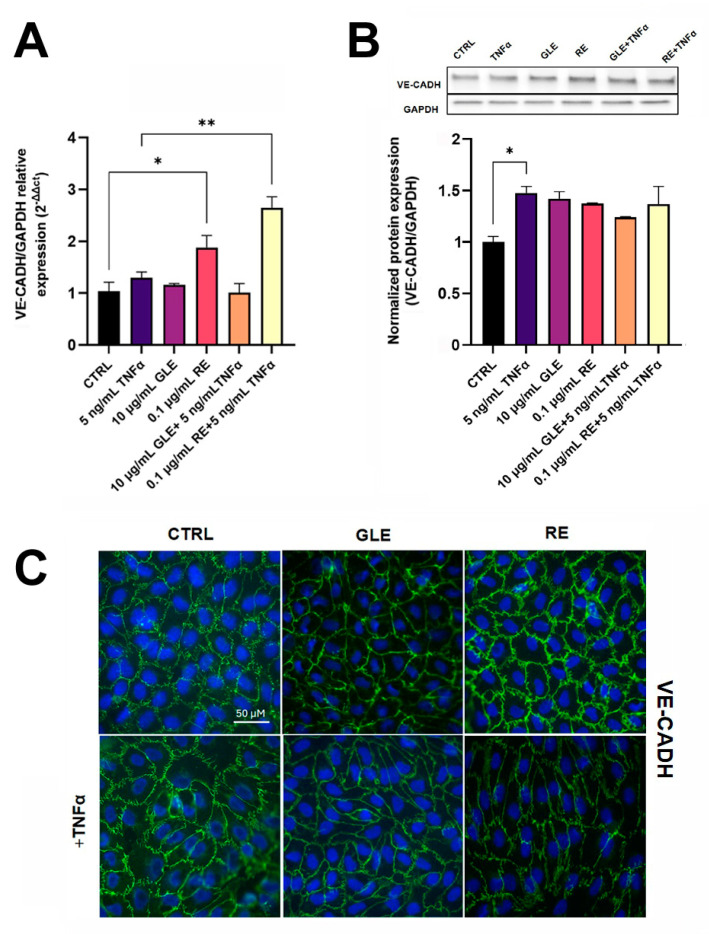
Effect of 10 µg/mL GLEs and 0.1 µg/mL REs, alone and in co-treatment with 5 ng/mL TNFα, on the expression and the localization of the AJ protein VE-CADHERIN in BLECs, determined by qRT-PCR (**A**), Western blot (**B**) and immunofluorescence microscopy with nuclear counterstaining using DAPI (**C**). The original Western Blot is included in the Supplementary Materials. Each bar is representative of at least three independent experiments performed in triplicate. Values are expressed as mean fold change ± SEM compared to the control. One-way ANOVA followed by Tukey’s multiple comparison post test was used. * *p* < 0.05; ** *p* < 0.01.

Dealing with VE-CADHERIN, its mRNA level was not significantly different from that of the control in all experimental conditions, except for the treatment with REs and the co-treatment with REs and TNFα, which showed an increase by about 1.87- and 2.35-fold compared to the control and TNFα alone, respectively (Figure 8A). On the other hand, Western blot analysis showed an upregulation of VE-CADHERIN protein in all experimental conditions, compared to the control (Figure 8B). More interestingly, the results of VE-CADHERIN immunolocalization (Figure 8C) were similar to those of CLAUDIN-5. In fact, exposure to TNFα caused delocalization of the protein, and after co-treatment with GLEs or REs, also VE-CADHERIN appeared to return to cell boundaries. Of note is that all the microscopic observations showed that, although co-treatment was able to re-establish the mechanical integrity of the barrier-forming layer, the apparent changes in cell morphology observed in the presence of TNFα still persisted. These cumulative results confirmed the active role of the extracts in safeguarding the architectural cohesion of the barrier under inflammatory conditions.

## 4. Discussion

### 4.1. Anti-Inflammatory Effects of P. oceanica’s Extracts: Modulation of NO and IL-6

The objective of this study was to assess the potential protective effects of *P. oceanica*’s extracts, particularly those sourced from its green leaves (GLEs) and rhizomes (REs), on the BBB in the presence of inflammatory conditions. Since neuroinflammation is a key trigger of BBB dysfunction, we first explored how these extracts modulate key inflammatory mediators.

Our results showed that co-treatment with GLEs or REs significantly reduced TNFα-induced NO production, indicating that the extracts exert anti-inflammatory effects at the endothelial level. Interestingly, IL-6 levels remained elevated despite extract treatment. While IL-6 is traditionally viewed as a pro-inflammatory cytokine that compromises barrier function, its role at the BBB is more nuanced. For example, Cohen et al. (2014) reported the occurrence of dose-dependent effects, where lower IL-6 concentrations actually promoted CLAUDIN-5 expression in ovine cerebral microvessels [36]. Thus, the persistent IL-6 expression in our model does not necessarily contradict the observed protective effects of GLEs and REs. Future studies using IL-6 knockdown or neutralization strategies may help clarify its context-dependent actions on BBB integrity.

### 4.2. Inhibition of NLRP3 Inflammasome Activation

Further supporting the anti-inflammatory properties of the extracts, we observed TNFα activating the NLRP3 inflammasome in brain ECs, a key step that initiates inflammatory cascades and contributes to BBB breakdown. Co-treatment with GLEs or REs effectively inhibited this activation, suggesting their ability to interfere with inflammasome signaling pathways. This observation aligns with previous findings showing that polyphenolic compounds can suppress NLRP3 activation in endothelial model systems [37,38,39]. Notably, while our model demonstrated inflammasome activation upon TNFα exposure alone, other studies have shown that a secondary stimulus (e.g., ATP or oxidative stress) may be necessary depending on the specific system used [40,41]. This underlines the cell-type and context specificity of inflammasome dynamics and suggests that our in vitro model captures an early and sensitive phase of inflammatory responses in ECs.

### 4.3. Regulation of Adhesion Molecules and Immune Cell Recruitment

The anti-inflammatory effects of the extracts were also evident in their impact on adhesion molecules. TNFα upregulated ICAM-1 and VCAM-1 expression, both of which are central for immune cell adhesion and transmigration during neuroinflammatory responses [42]. GLEs and REs significantly reduced this upregulation, indicating their ability to prevent immune-endothelial cell interactions that exacerbate BBB permeability. These findings reinforce the notion that *P. oceanica*’s extracts help maintain endothelial quiescence and limit leukocyte recruitment.

### 4.4. Preservation of BBB Integrity Through Junctional Protein Regulation

One of the most critical observations in our study was the preservation of junctional protein expression and localization following extract treatment. TNFα exposure disrupted BBB integrity by decreasing CLAUDIN-5 levels and delocalizing both CLAUDIN-5 and VE-CADHERIN from the cell–cell contacts to the cytoplasm. These events are consistent with established models of inflammatory breakdown of the BBB [21,43,44]. Importantly, co-treatment with GLEs or REs partially reversed these effects, restoring the proper localization of junctional proteins. This finding is particularly relevant because the maintenance of TJ and AJ complexes is fundamental for the stability of the BBB. The observed stabilization of VE-CADHERIN and CLAUDIN-5 echoes previous studies highlighting their role as critical regulators of BBB integrity under inflammatory stress [45,46,47,48].

### 4.5. Bioactive Compounds in P. oceanica’s Extracts and Their Mechanisms

The protective effects of the extracts are likely due to the presence of multiple bioactive polyphenols and antioxidant compounds. As characterized by Abruscato et al. [6], GLEs contain high levels of caffeic acid methyl ester (CAME), while REs are rich in delphinidin-3-glucoside, quercetin-3-O-galactoside, procyanidin dimer B type isomer 2 and 3, vanillic acid, and other polyphenols such as epicatechin, myricetin, ellagic acid, and resveratrol in trace amounts. CAME is known to activate the HO-1/Nrf2 pathway, promoting endothelial resilience against oxidative and inflammatory stress [49]. Delphinidin-3-glucoside improves the viability endothelial cells and reduces their permeability [50]. Quercetin-3-O-galactoside (hyperoside) inhibits TLR4/NF-κB signaling and upregulates junctional proteins in response to pro-inflammatory insults [51,52]. Other compounds, such as vanillic acid, epicatechin, myricetin, ellagic acid, and resveratrol, may contribute to providing additional anti-inflammatory and antioxidant benefits [53,54,55,56,57,58,59,60], including the reduction of ROS release and the preservation of junctional protein expression. The synergistic action of these molecules likely underlies the overall protective phenotype observed in our model system.

### 4.6. Suitability and Limitations of the In Vitro BBB Model

While our in vitro static BBB model does not replicate the full complexity of the neurovascular unit, including shear stress and immune cell dynamics, it remains a well-validated tool for assessing endothelial cell responses. Moreover, since BBB formation occurs predominantly at the capillary level where the flow is minimal, the lack of shear stress is unlikely to invalidate our conclusions. Our previous work with this model, including monocyte transmigration assays [35], supports its relevance for the preliminary screening of barrier-modulating compounds.

## 5. Conclusions

In conclusion, our study highlights the promising role of *P. oceanica*’s extracts as natural agents capable of modulating the inflammatory responses and preserving the structural and functional integrity of the BBB. Their protective effects appear to be mediated by the stabilization of tight and adherens junctions, suggesting potential applications in the prevention and treatment of neuroinflammatory disorders associated with BBB dysfunction. The identification of specific bioactive compounds further reinforces the value of marine biodiversity as a source of novel neuroprotective agents. However, the clinical relevance of these findings remains to be established. Additional in vivo studies are needed to confirm the protective effects of the extracts on the BBB, explore their pharmacokinetics and tissue distribution, particularly their ability to reach the brain after oral or systemic administration, and to elucidate their mechanisms of action and therapeutic efficacy in relevant disease models.

## Figures and Tables

**Table 1:** Main polyphenolic components of GLEs and REs determined through a qualitative–quantitative approach with HPLC. n.q: not quantifiable [2].

Polyphenol	GLEs (µg/g)	REs (µg/g)
Delphinidin-3-glucoside	n.q	11.52
Quercetin 3-O-galactoside	n.q	10.81
Procyanidin dimer B type isomer 2	n.q	0.20
Procyanidin dimer B type isomer 3	n.q	0.30
Vanillic acid		0.6
Caffeic acid methyl ester	0.37	-

## Data Availability

All data generated or analyzed during this study are included in this published article.

## Supplementary Materials

The following supporting information can be downloaded at: https://www.mdpi.com/article/10.3390/biology14060699/s1.
