# Supplementary material for: Protective Effects of Extracts from Green Leaves and Rhizomes of Posidonia oceanica (L.) Delile on an In Vitro Model of the Human Blood–Brain Barrier
Source: Biology (Basel). 2025 Jun 14;14(6):699. doi: 10.3390/biology14060699 (PMC12189290; doi:10.3390/biology14060699)
Supplement: Supplementary file 1 [file biology-14-00699-s001.zip › biology-3676096-supplementary.pdf]

NLRP3 (Ab263899) AND βACTIN

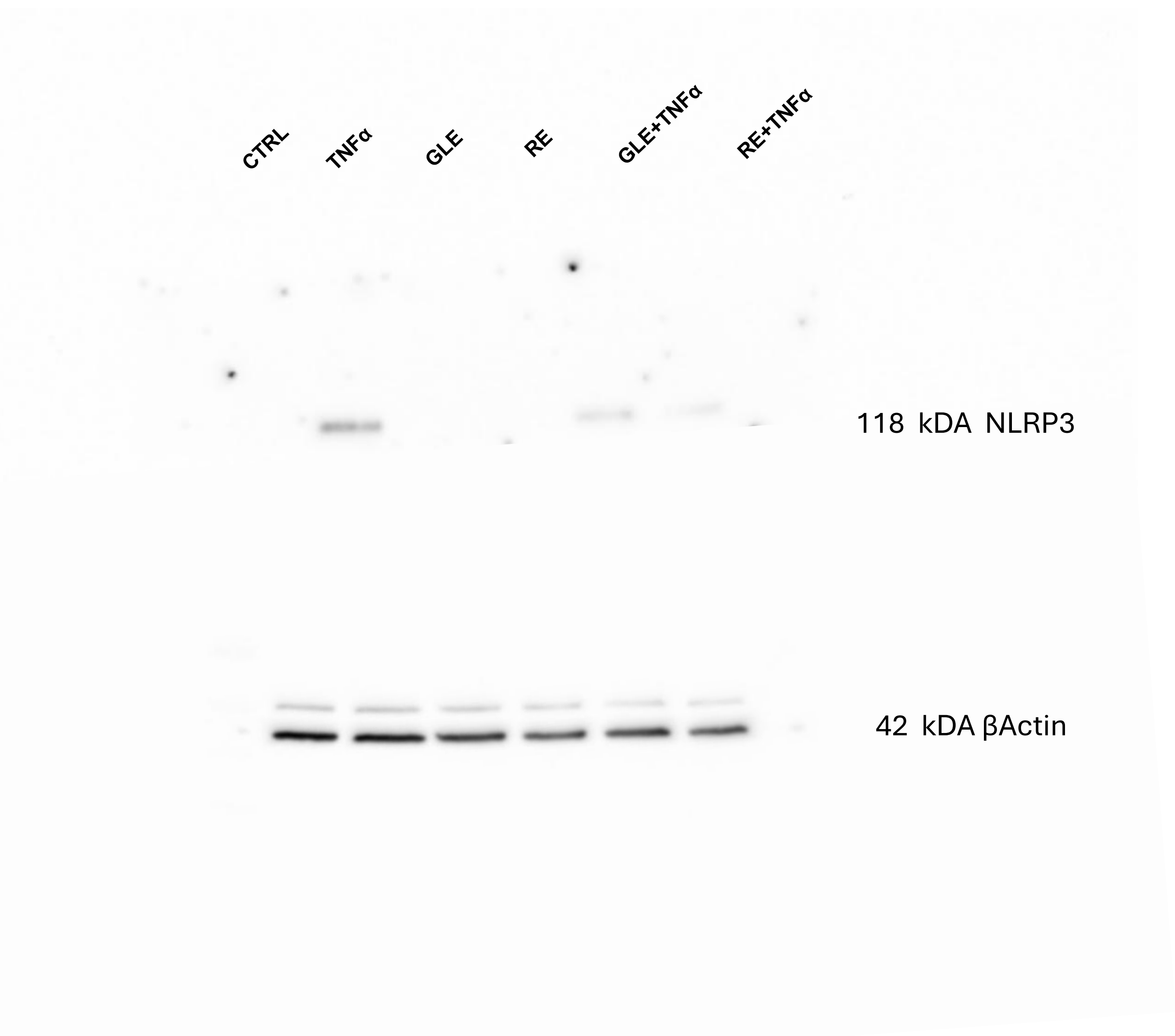

Protein marker: Bio Rad- Precision Plus Protein All Blue Standards

Complete membrane for NLRP3 and βactin bands merged with molecular weight marker (Membrane cut between 100 and 75 kDa)

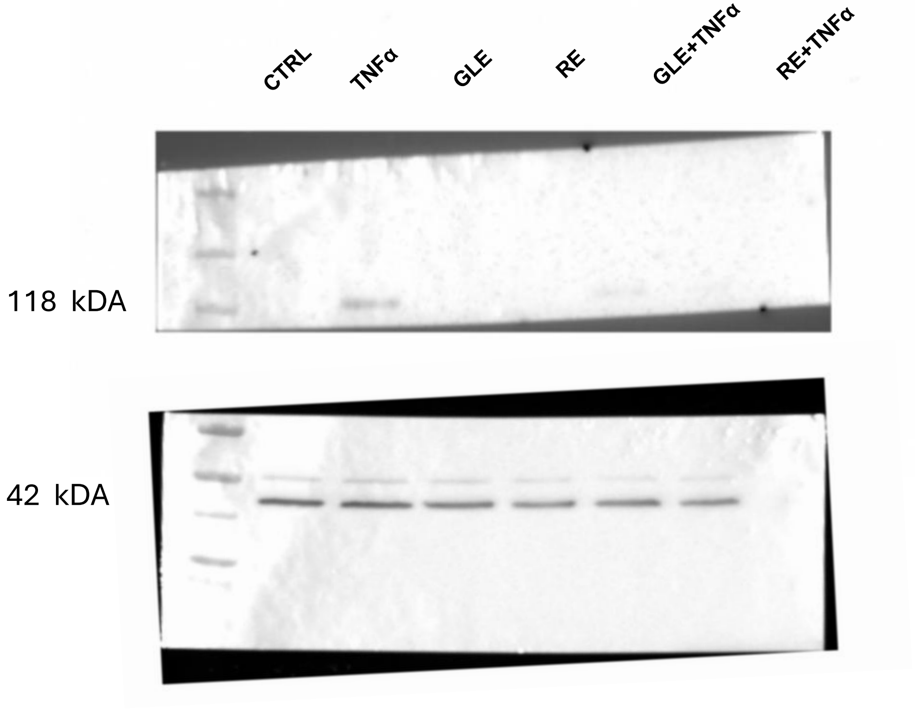

Lane 1. Ctrl
Lane 2. 5ng/mL TNFα
Lane 3. 10 μg/mL GLE
Lane 4. 0.1 μg/mL RE
Lane 5. 10 μg/mL GLE+ 5ng/mL TNFα
Lane.6 0.1 μg/mL RE+ 5ng/mL TNFα

VCAM-1(PA5-80213) AND GAPDH

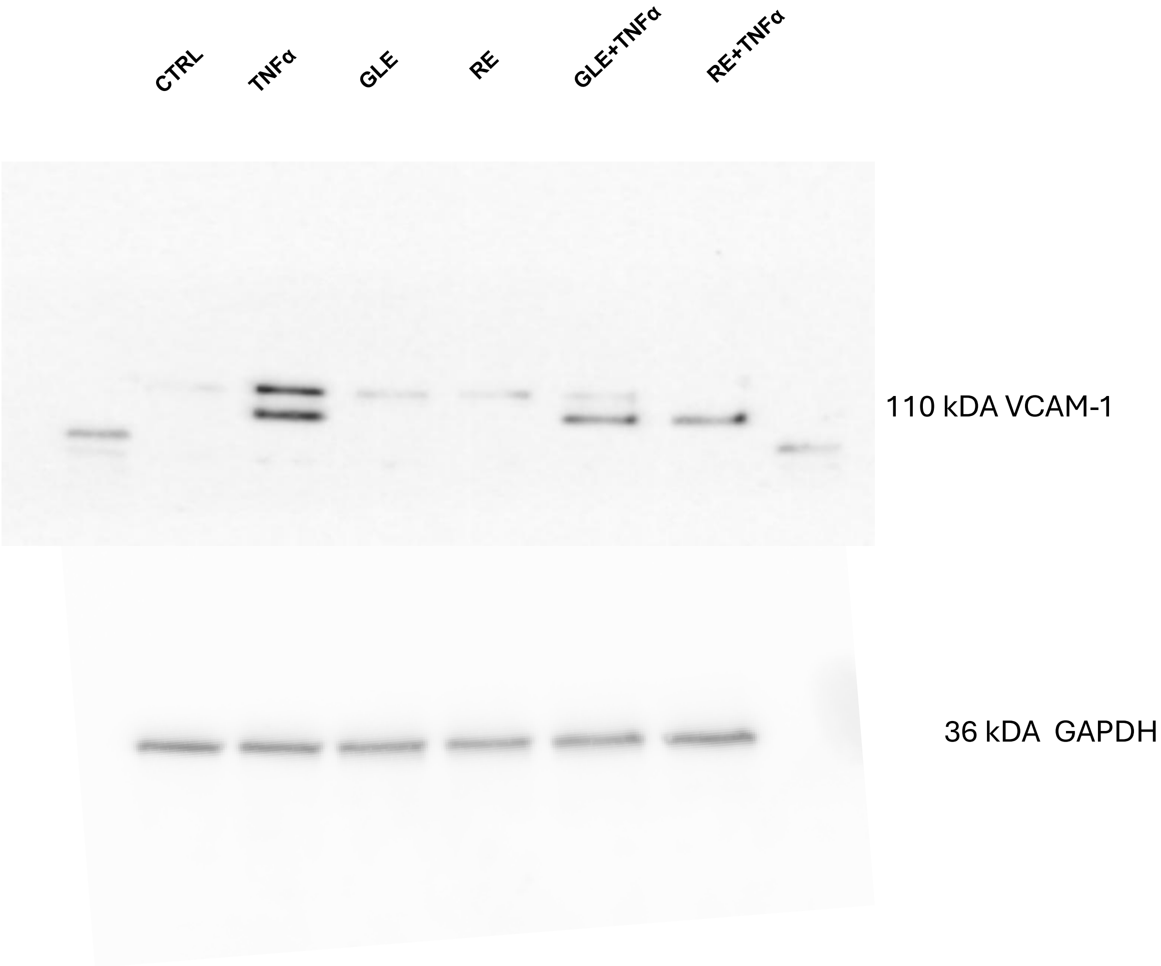

Protein marker: Bio Rad- Precision Plus Protein All Blue Standards

Complete membrane for VCAM-1 and GAPDH bands merged with molecular weight marker (Membrane cut between 50 and 37 kDa)

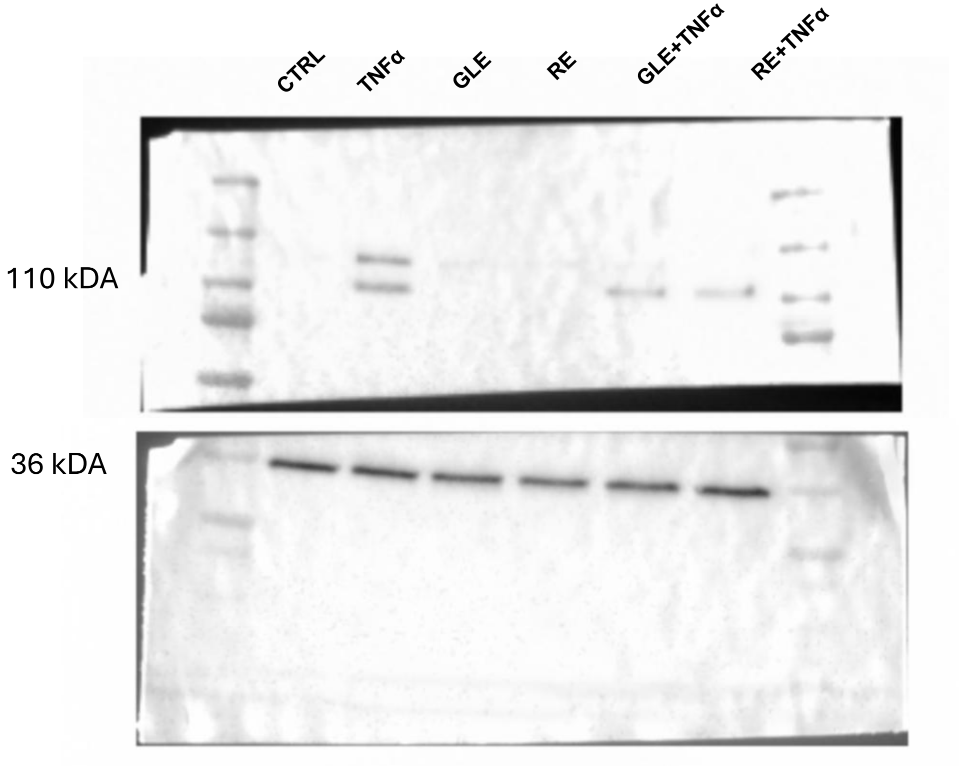

Lane 1. Ctrl
Lane 2. 5ng/mL TNFα
Lane 3. 10 µg/mL GLE
Lane 4. 0.1 µg/mL RE
Lane 5. 10 µg/mL GLE+ 5ng/mL TNFα
Lane.6 0.1 µg/mL RE+ 5ng/mL TNFα

ICAM-1 (Ab53013) AND βACTIN

ICAM-1 bands merged with molecular weight marker

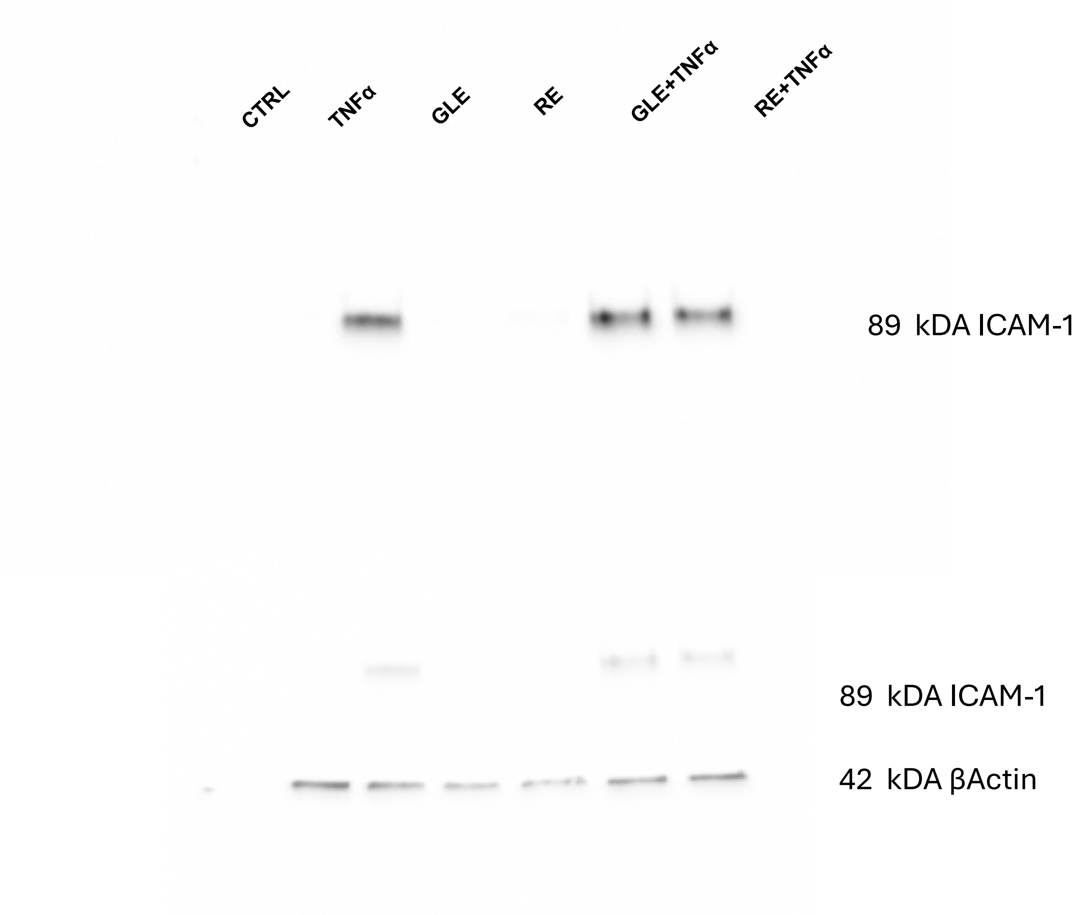

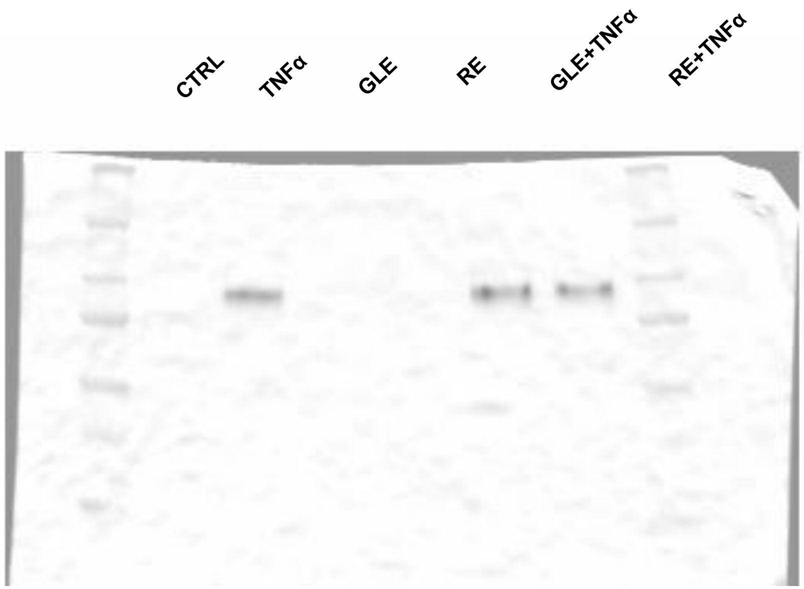

Lane 1. Ctrl
Lane 2. 5ng/mL TNFα
Lane 3. 10 μg/mL GLE
Lane 4. 0.1 μg/mL RE
Lane 5. 10 μg/mL GLE+ 5ng/mL TNFα
Lane.6 0.1 μg/mL RE+ 5ng/mL TNFα

The antibodies were incubated in the same membrane. First ICAM-1 and then βactin

Protein marker: Bio Rad- Precision Plus Protein All Blue Standards

CLAUDIN-5(GTX49370) AND βACTIN

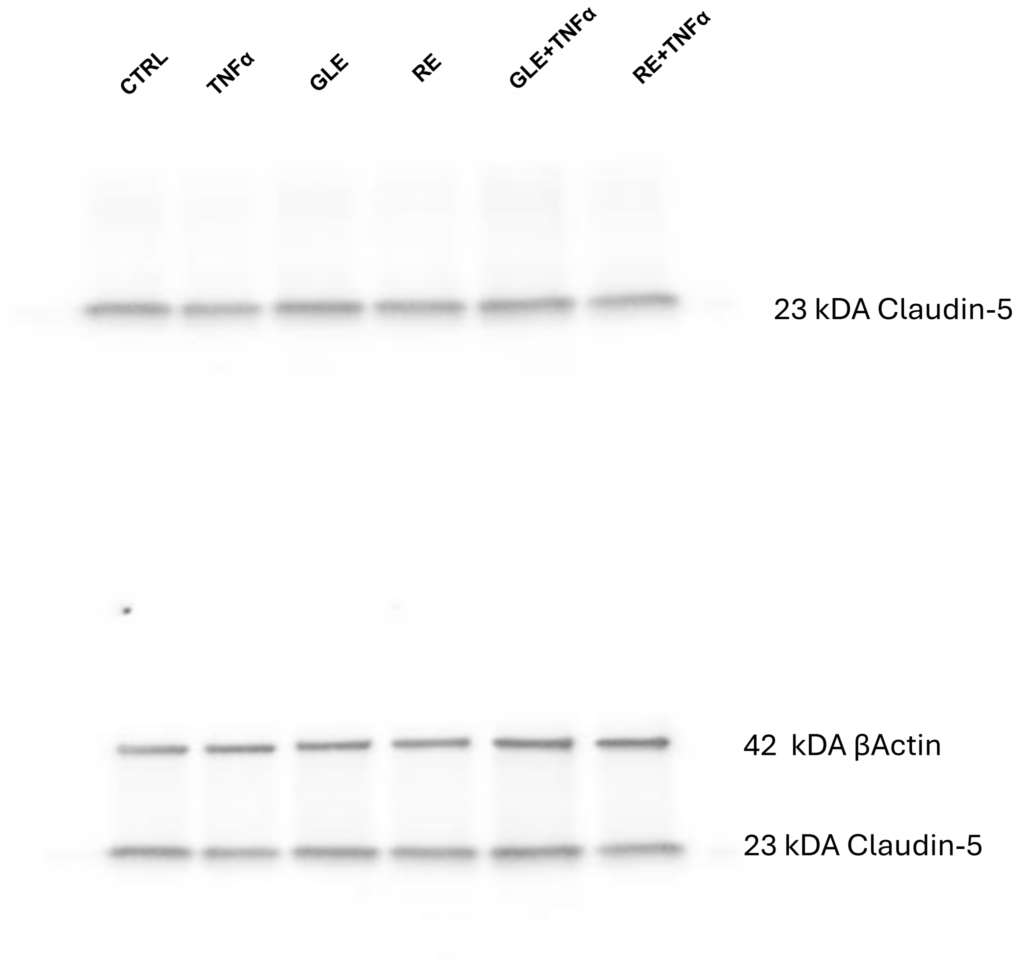

Complete membrane for Claudin-5 and βactin bands merged with molecular weight marker (Membrane cut at 50 kDa)

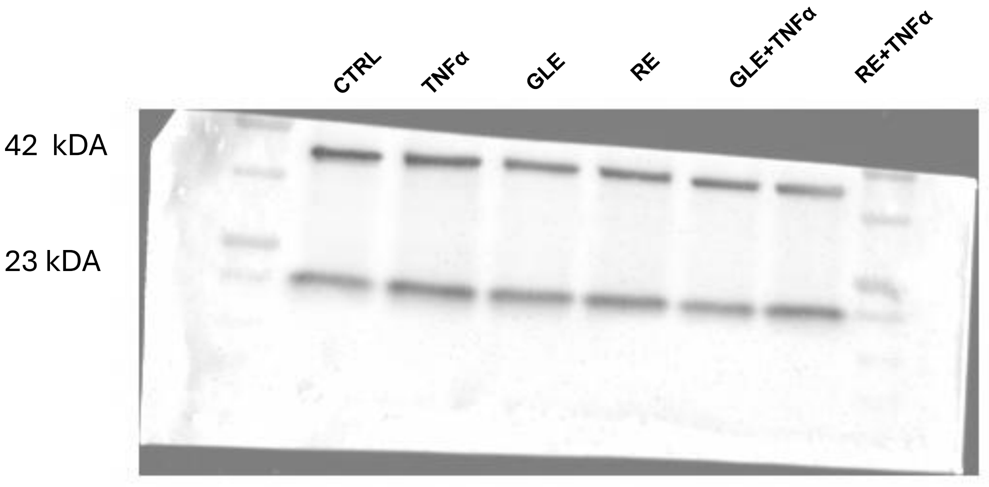

Lane 1. Ctrl
Lane 2. 5ng/mL TNFα
Lane 3. 10 µg/mL GLE
Lane 4. 0.1 µg/mL RE
Lane 5. 10 µg/mL GLE+ 5ng/mL TNFα
Lane.6 0.1 µg/mL RE+ 5ng/mL TNFα

Protein marker: Bio Rad- Precision Plus Protein All Blue Standards

VE-CADH (Ab33168) AND GAPDH

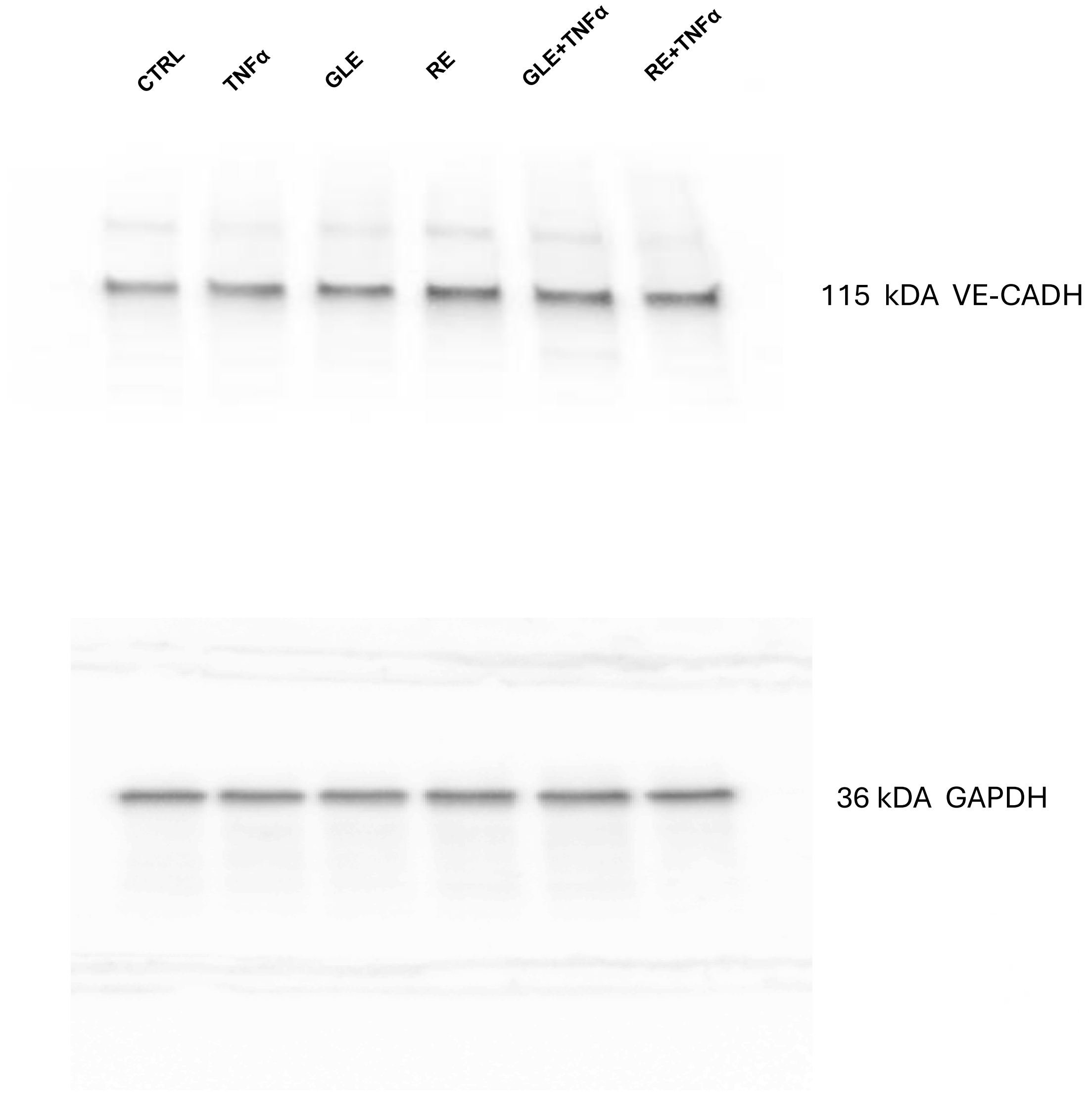

Protein marker: Bio Rad- Precision Plus Protein All Blue Standards

Complete membrane for Ve-Cadh and GAPDH bands merged with molecular weight marker (Membrane cut at 50 kDa)

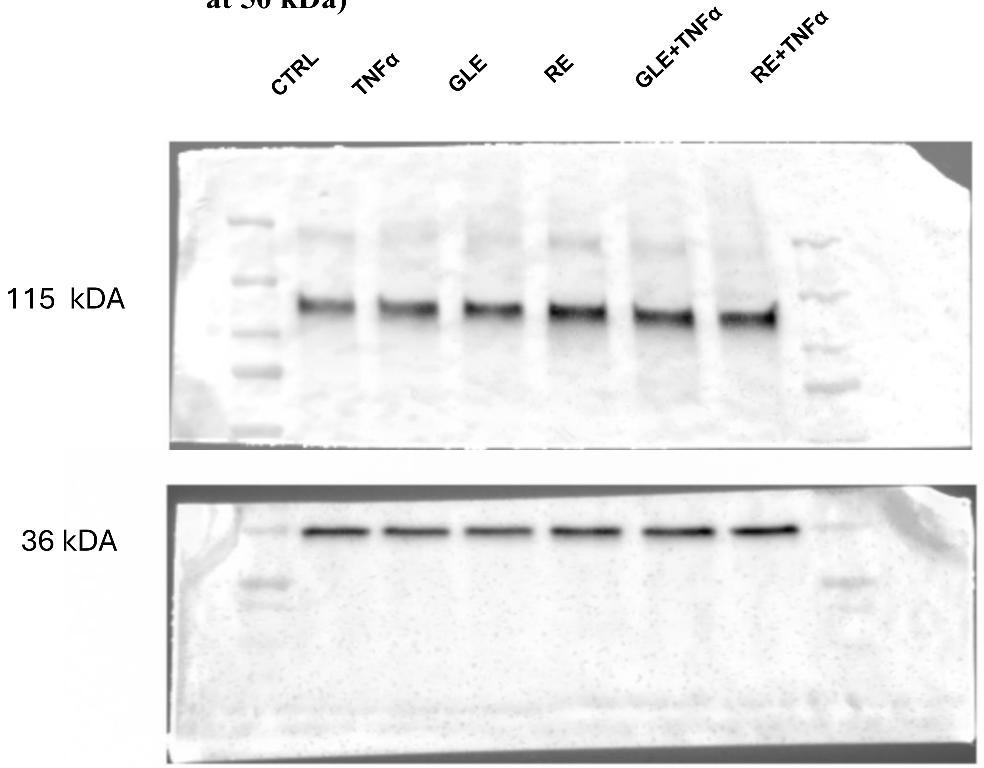

Lane 1. Ctrl
Lane 2. 5ng/mL TNFα
Lane 3. 10 µg/mL GLE
Lane 4. 0.1 µg/mL RE
Lane 5. 10 µg/mL GLE+ 5ng/mL TNFα
Lane.6 0.1 µg/mL RE+ 5ng/mL TNFα
